# Next-generation sequencing reveals novel differentially regulated mRNAs, lncRNAs, miRNAs, sdRNAs and a piRNA in pancreatic cancer

**DOI:** 10.1186/s12943-015-0358-5

**Published:** 2015-04-25

**Authors:** Sören Müller, Susanne Raulefs, Philipp Bruns, Fabian Afonso-Grunz, Anne Plötner, Rolf Thermann, Carsten Jäger, Anna Melissa Schlitter, Bo Kong, Ivonne Regel, W Kurt Roth, Björn Rotter, Klaus Hoffmeier, Günter Kahl, Ina Koch, Fabian J Theis, Jörg Kleeff, Peter Winter, Christoph W Michalski

**Affiliations:** Molecular BioSciences, Goethe University, Frankfurt am Main, Germany; GenXPro GmbH, Frankfurt Biotechnology Innovation Center, Frankfurt am Main, Germany; Department of Surgery, Klinikum Rechts der Isar, Technische Universität München, Munich, Germany; Molecular Bioinformatics Group, Institute of Computer Science, Cluster of Excellence Frankfurt ‘Macromolecular Complexes’ Faculty of Computer Science and Mathematics, Frankfurt am Main, Germany; Department of Surgery, University of Heidelberg, Heidelberg, Germany; GFE Blut mbH, Frankfurt Biotechnology Innovation Center, Frankfurt am Main, Germany; Institute of Computational Biology, Helmholtz Zentrum Munich, Neuherberg, Germany; Department of Mathematics, TU Munich, Boltzmannstrasse 3, Garching, Germany; Department of Pathology, Klinikum Rechts der Isar, Technische Universität München, Munich, Germany

**Keywords:** Pancreatic cancer, MACE, 3′UTR, miRNA, Long non-coding RNA, Wnt signalling, Next-generation sequencing, ZEB1, TCF4

## Abstract

**Background:**

Previous studies identified microRNAs (miRNAs) and messenger RNAs with significantly different expression between normal pancreas and pancreatic cancer (PDAC) tissues. Due to technological limitations of microarrays and real-time PCR systems these studies focused on a fixed set of targets. Expression of other RNA classes such as long intergenic non-coding RNAs or sno-derived RNAs has rarely been examined in pancreatic cancer. Here, we analysed the coding and non-coding transcriptome of six PDAC and five control tissues using next-generation sequencing.

**Results:**

Besides the confirmation of several deregulated mRNAs and miRNAs, miRNAs without previous implication in PDAC were detected: miR-802, miR-2114 or miR-561. SnoRNA-derived RNAs (e.g. sno-HBII-296B) and piR-017061, a piwi-interacting RNA, were found to be differentially expressed between PDAC and control tissues. *In silico* target analysis of miR-802 revealed potential binding sites in the 3′ UTR of *TCF4*, encoding a transcription factor that controls Wnt signalling genes. Overexpression of miR-802 in MiaPaCa pancreatic cancer cells reduced TCF4 protein levels. Using Massive Analysis of cDNA Ends (MACE) we identified differential expression of 43 lincRNAs, long intergenic non-coding RNAs, e.g. LINC00261 and LINC00152 as well as several natural antisense transcripts like HNF1A-AS1 and AFAP1-AS1. Differential expression was confirmed by qPCR on the mRNA/miRNA/lincRNA level and by immunohistochemistry on the protein level.

**Conclusions:**

Here, we report a novel lncRNA, sncRNA and mRNA signature of PDAC. *In silico* prediction of ncRNA targets allowed for assigning potential functions to differentially regulated RNAs.

**Electronic supplementary material:**

The online version of this article (doi:10.1186/s12943-015-0358-5) contains supplementary material, which is available to authorized users.

## Background

A five year survival of around 4% makes pancreatic ductal adenocarcinoma (PDAC) the fourth leading cause of cancer-related deaths worldwide [1]. To better understand the aggressive growth and the poor response of PDAC to chemotherapeutic agents, studies are required that focus on molecular mechanisms underlying pancreatic cancer development and progression [2].

Somatic mutations, alterations in coding- and non-coding RNA expression as well as in the methylome of pancreatic cancer cells have been intensively studied [3]. The application of NGS technologies such as RNA-seq, whole exome sequencing or bisulfite sequencing to PDAC samples provided an unbiased view on genetic and epigenetic alterations [4-6]. However, studies investigating the small ncRNAome of PDAC tissues and healthy pancreas utilizing NGS methods are rare.

Studies utilizing microarrays revealed that deregulated microRNAs (miRNAs) have an impact on coding-gene expression in PDAC [7-12]. MiRNAs are a class of small non-coding RNAs (sncRNAs) that can repress gene expression [13]. Due to the known technical limitations of microarrays or real-time PCR most studies were focused on a fixed set of miRNA targets and many other sncRNA types have not been implicated in PDAC, but their deregulation and contribution to cancer progression has been described for other cancer types [14,15]. These sncRNAs include small nucleolar-derived RNAs (sno-derived RNAs, sdRNAs), functioning like miRNAs, or regulating splicing and translation [16], as well as piwi-interacting RNAs (piRNAs), that are associated with chromatin organization, messenger RNA stability and genome structure [17].

Similarly, only few studies have reported altered expression of long non-coding RNAs (lncRNAs) in PDAC [18]. LncRNAs represent a diverse class of modestly conserved, polyadenylated, non-protein-coding RNAs with essential roles in tumorigenesis [19]. LncRNAs comprise, among others, long intergenic non-coding RNAs (lincRNAs) and natural antisense transcripts (NATs). NATs, which are transcribed from the opposite (“anti-sense”) strand of a protein-coding gene can either stabilize or destabilize the expression of their sense partner [20], lincRNAs act as competitive endogenous RNAs (ceRNAs) and sequester miRNAs (“miRNA sponge”) [21], target chromatin modification complexes or RNA-binding proteins to alter gene expressing programs [22].

Here, we used high-throughput NGS-based technologies, namely Massive Analysis of cDNA Ends (MACE) and small RNA-sequencing (sRNA-seq), to characterize the complete coding- and non-coding transcriptomes of tissues from six PDAC patients and five normal controls. We merged the expression pattern obtained by the two techniques to gain insights into the altered miRNA regulation of coding gene expression in PDAC as compared to normal pancreatic tissues. Additionally, we provide evidence for differential expression of a piRNA and several sdRNAs, lincRNAs and NATs in PDAC.

## Results

Differences in the coding transcriptome, lncRNAs and sncRNAs between pancreatic cancer tissue from six patients and normal pancreatic tissue from five controls were assessed by MACE and sRNA-seq. Sequencing results were experimentally validated by quantitative real time PCR (qPCR) for seven genes (*CD1A, CTHRC1, FOXL1, GPR87, KLK7, TCF4, ZEB1*), four miRNAs (miR-103a-3p, 135b-5p, 145-5p, 802) and two lincRNAs (LINC00152, LINC00261).

**Figure 1:**
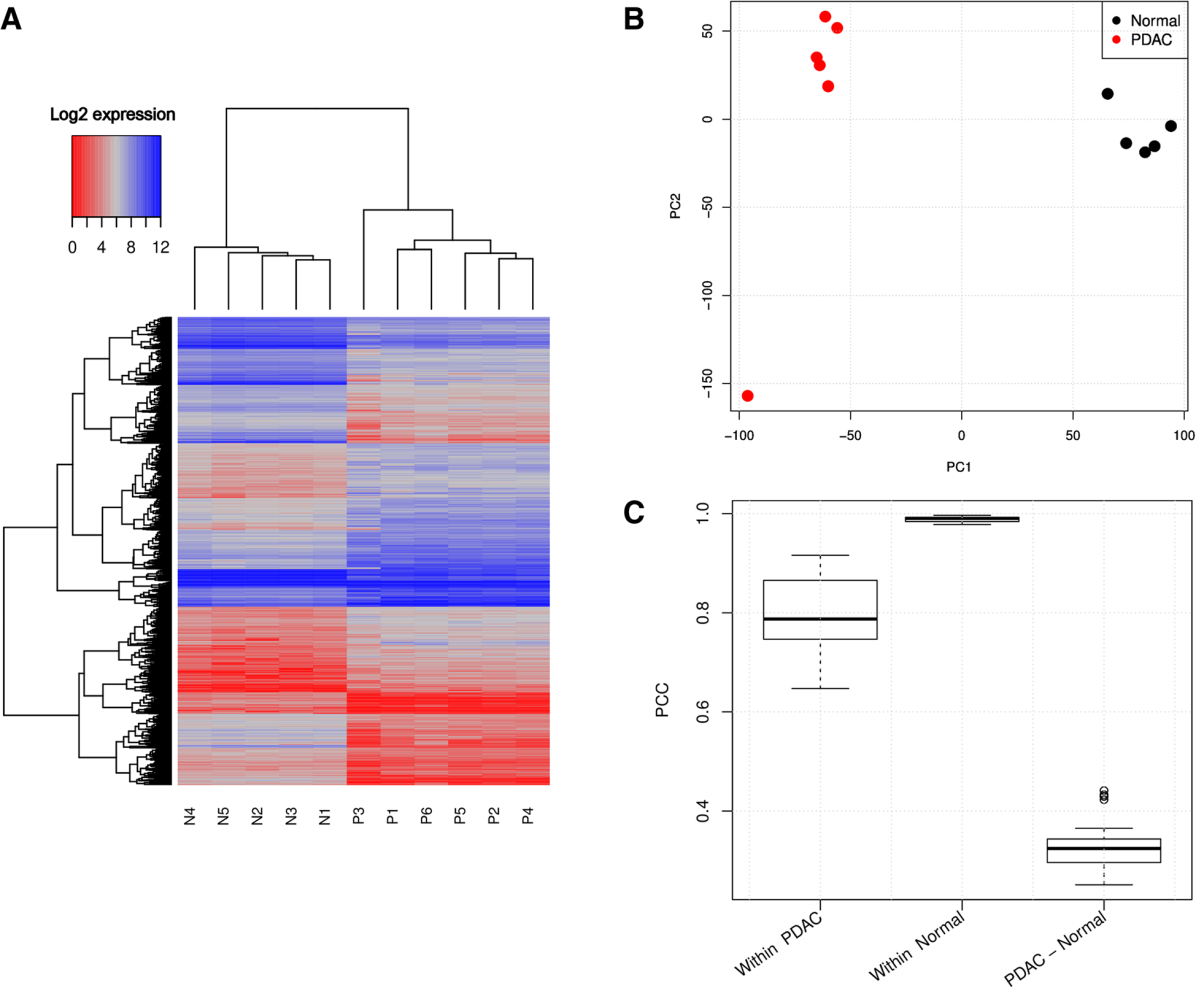
NGS MACE profiles discriminate PDAC from healthy control tissues. **A)** Unsupervised hierarchical cluster analysis of differentially expressed genes with euclidean distance measure clearly separates healthy controls (N) and diseased patients (P). **(B)** Principle component analysis (PCA) of all genes from the eleven samples under scrutiny. The first (x-axis) and second principal component (y-axis) account for 32% and 18%, respectively, of the total variation in the data. **(C)** Pearson product–moment correlation coefficient (PCC) for all samples compared within the control and patient group as well as between both groups.

Overall, 396,542,460 sequences were generated. The amount of high-quality reads used for the analysis ranged from 4,150,706 reads in the MACE library for cancer patient P3 to 11,007,400 in the sRNA-seq normal pancreas library N2 (see Table 1). Robust expression was detected for 13,614 coding genes and 432 lncRNAs (Additional file 1: Table S1) whereas 1,961 mRNAs and 43 lncRNAs of these were significantly differentially expressed between cancer and control tissues (Additional file 2: Table S2). Unsupervised hierarchical clustering, principal component analysis (PCA) and Pearson’s moment product correlation coefficients (PCCs) revealed a clear separation between the groups based on the sequencing results (Figure 1A-C). Notably, 70% of all reads across control libraries mapped to 25 genes (*PNLIPRP1, CELA3B, CPA2, CTRL, GP2, CPB1, CTRC, RBPJL, KLK1, PLA2G1B, CELA2A, CEL, GNMT, CELA3A, CPA1, PRSS3P2, PRSS3, CLPS, PNLIP, SLC39A5, SPINK1, CTRB1, TMED11P, PRSS1, GATM*), encoding pancreatic acinar cell secretory- and related proteins (Table 2). In cancer libraries, only 3% of reads were annotated to these genes. Their homogenous, robust expression across all controls and strong downregulation in all PDACs underlines the existence of normal pancreas function in control libraries, which is lost in PDAC tissues. The significantly up-/downregulated lncRNAs, as determined by MACE, are listed in Table 3.

**Table 1:** **MACE and sRNA-seq read statistics**

**a) MACE**					
**Condition**	**Sample**	**Raw Reads**	**PCR-Duplicate removed**	**Region of interest**	**Sign. Upreg. RNAs**
	N1	20,406,430	11,609,003	7,811,603	
	N2	13,749,503	8,791,698	6,033,792	
Normal	N3	17,404,996	10,701,183	7,024,298	963
	N4	14,868,474	9,666,314	6,411,998	
	N5	18,213,260	10,782,506	7,353,927	
	P1	8,706,841	7,921,368	4,377,691	
	P2	9,940,175	8,802,579	4,250,025	1,041
PDAC	P3	8,463,912	7,489,546	4,150,706	
	P4	8,438,058	7,612,616	4,422,792	
	P5	13,350,594	11,634,367	5,405,594	
	P6	9,218,757	8,161,312	4,607,707	
**b) sRNA-seq**					
	N1	19,268,787	9,886,904	6,603,800	
	N2	33,372,333	15,945,439	11,007,400	
Normal	N3	22,217,672	11,571,686	7,773,570	45
	N4	27,924,894	6,763,260	5,439,290	
	N5	23,421,799	9,025,877	6,482,120	
	P1	24,349,755	9,837,308	6,887,790	
	P2	15,855,377	6,966,105	5,063,220	78
PDAC	P3	19,711,102	9,580,636	6,321,340	
	P4	25,889,624	10,404,479	7,344,910	
	P5	22,130,647	11,117,354	7,402,310	
	P6	19,639,470	8,440,144	5,964,730	

For each sample of control (N) and PDAC (P) tissues the number of raw sequencing reads, PCR duplicate-free reads, reads mapped to regions of interest (exons -MACE or small ncRNAs -sRNA-seq) and the number of deregulated RNAs is provided for MACE and sRNA-seq libraries, respectively.

**Table 2:** **Expression of genes encoding pancreatic acinar cell secretory- and related proteins**

	***PRSS1***	***CLPS***	***CTRB1***	***PNLIP***	***CPA1***	***CEL***	***CELA3A***	***PLA2G1B***	***CTRC***	***CELA2A***
N1	20.6	20.4	20.0	20.0	19.9	19.1	19.0	18.6	18.8	18.5
N2	20.8	20.3	20.3	20.2	19.9	19.2	19.2	18.8	18.3	18.5
N3	20.4	20.0	19.9	19.7	19.7	18.9	18.8	18.3	18.0	18.5
N4	20.1	19.5	19.8	19.3	19.7	18.6	18.3	18.0	18.3	17.3
N5	20.7	20.1	20.3	20.0	19.9	19.1	18.7	19.0	18.9	18.5
P1	13.4	12.8	12.8	12.4	12.0	11.2	11.2	10.4	10.3	10.3
P2	13.9	13.2	13.2	12.8	12.6	11.7	11.6	10.9	10.8	10.7
P3	14.4	13.3	14.1	13.5	13.4	12.4	12.1	11.6	11.8	10.9
P4	14.3	13.2	14.0	13.4	13.3	12.3	11.9	11.5	11.7	10.8
P5	13.8	13.2	13.2	12.8	12.5	11.6	11.6	10.8	10.7	10.7
P6	14.0	13.4	13.4	13.0	12.7	11.8	11.9	11.0	10.9	10.9
Mean N	20.6	20.1	20.1	19.9	19.8	19.0	18.8	18.6	18.5	18.3
Mean P	14.0	13.2	13.5	13.0	12.8	11.9	11.7	11.1	11.1	10.7
Log2 FC	6.4	6.9	6.6	6.8	7.0	7.1	7.1	7.5	7.3	7.6

The normalized logarithmic expression of the ten most highly expressed genes in normal pancreas tissues for all five control- (N) and six PDAC (P) sequencing libraries. All genes encode pancreas-specific proteins and are homogenously, highly expressed across all control libraries and more than ~100-fold decreased in all PDAC tissues.

**Table 3:** **Differentially expressed lncRNAs**

**lncRNA**	**NEV Normal**	**NEV PDAC**	**Log2 FC**	**FDR**	**Class**	**Regulation**	**PED**	**Other**
LINC00671	81.13	0.47	7.45	0.0000000	lincRNA	DOWN		
DGCR5	83.72	2.89	4.86	0.0000000	lncRNA	DOWN		
LINC00261	278.16	7.04	5.30	0.0000000	lincRNA	DOWN		D [24]
TRHDE-AS1	24.54	1.05	4.54	0.0000015	NAT	DOWN		
PITPNA-AS1	94.22	16.50	2.51	0.0000085	NAT	DOWN		
lincRNA	820.02	190.34	2.11	0.0000189	ncRNA	DOWN		
SNHG9	595.61	149.36	2.00	0.0000217	lincRNA	DOWN		
lincRNA	27.79	134.36	−2.27	0.0000230	ncRNA	UP		U [24]
CEBPA-AS1	37.03	5.21	2.83	0.0000629	NAT	DOWN		
lincRNA	357.77	90.05	1.99	0.0000661	ncRNA	DOWN		
SCARNA22	56.28	7.07	2.99	0.0001779	ncRNA	DOWN		
SCARNA2	27.72	4.21	2.72	0.0002533	ncRNA	DOWN		
HNF1A-AS1	72.29	10.99	2.72	0.0002943	NAT	DOWN		U [26]
SDCBP2-AS1	18.87	1.90	3.31	0.0002948	NAT	DOWN		
NAT	23.83	3.16	2.91	0.0003537	ncRNA	DOWN		
NAT	11.60	56.22	−2.28	0.0003752	ncRNA	UP	U	
LINC00339	355.98	10.67	5.06	0.0004037	lincRNA	DOWN		
TMEM44-AS1	36.53	7.99	2.19	0.0009294	NAT	DOWN		
LINC00263	44.69	10.62	2.07	0.0010579	lincRNA	DOWN		
lncRNA	10.01	46.87	−2.23	0.0011936	ncRNA	UP	U	
NAPA-AS1	25.94	4.71	2.46	0.0012669	NAT	DOWN		
LINC00340	4.16	28.42	−2.77	0.0013636	lincRNA	UP		
EPB41L4A-AS1	303.58	97.66	1.64	0.0015839	NAT	DOWN		
SERTAD4-AS1	3.19	22.21	−2.80	0.0016944	NAT	UP		
LHFPL3-AS2	20.61	3.08	2.74	0.0023207	NAT	DOWN		
LINC00673	8.59	42.30	−2.30	0.0023447	lincRNA	UP		
DNAJC27-AS1	20.43	3.65	2.49	0.0030115	NAT	DOWN		
lncRNA	6.87	43.83	−2.67	0.0056448	ncRNA	UP	U	
BOLA3-AS1	34.96	9.42	1.89	0.0074620	NAT	DOWN		
ZNF503-AS2	21.89	4.65	2.24	0.0079046	NAT	DOWN		
ACVR2B-AS1	13.06	1.46	3.16	0.0095731	NAT	DOWN		
PRRT3-AS1	28.36	7.04	2.01	0.0095911	NAT	DOWN		
LINC00346	1.41	13.14	−3.22	0.0121525	lincRNA	UP		
LINC00086	11.86	1.76	2.76	0.0132691	lincRNA	DOWN		
RUNX1-IT1	2.57	30.23	−3.56	0.0136106	ncRNA	UP		
THAP7-AS1	29.29	8.11	1.85	0.0198907	NAT	DOWN		
ZNF503-AS1	11.56	1.93	2.58	0.0240067	NAT	DOWN		
RNF157-AS1	10.82	1.91	2.50	0.0245309	NAT	DOWN		
AFAP1-AS1	17.34	153.08	−3.14	0.0265179	NAT	UP		U [25]
PSMG3-AS1	24.17	6.92	1.80	0.0291786	NAT	DOWN		
LINC00578	2.50	13.79	−2.46	0.0404404	lincRNA	UP		
lncRNA	2,303.62	721.52	1.67	0.0452843	ncRNA	DOWN	D	
lncRNA	964.77	471.70	1.03	0.0464071	ncRNA	DOWN		

For each differentially expressed lncRNA (FDR < 0.05) the mean normalized expression values (NEV) for each group is provided together with the ratio between normal and PDAC expression in log2 scale (log2 FC). Furthermore, “Class” indicates the lncRNA type, “PED” the expression as provided by the pancreatic cancer expression database (U: Upregulated in PDAC, D: Downregulated in PDAC) and the last column presents information about the expression level of the RNA in other types of cancer.

**Figure 2:**
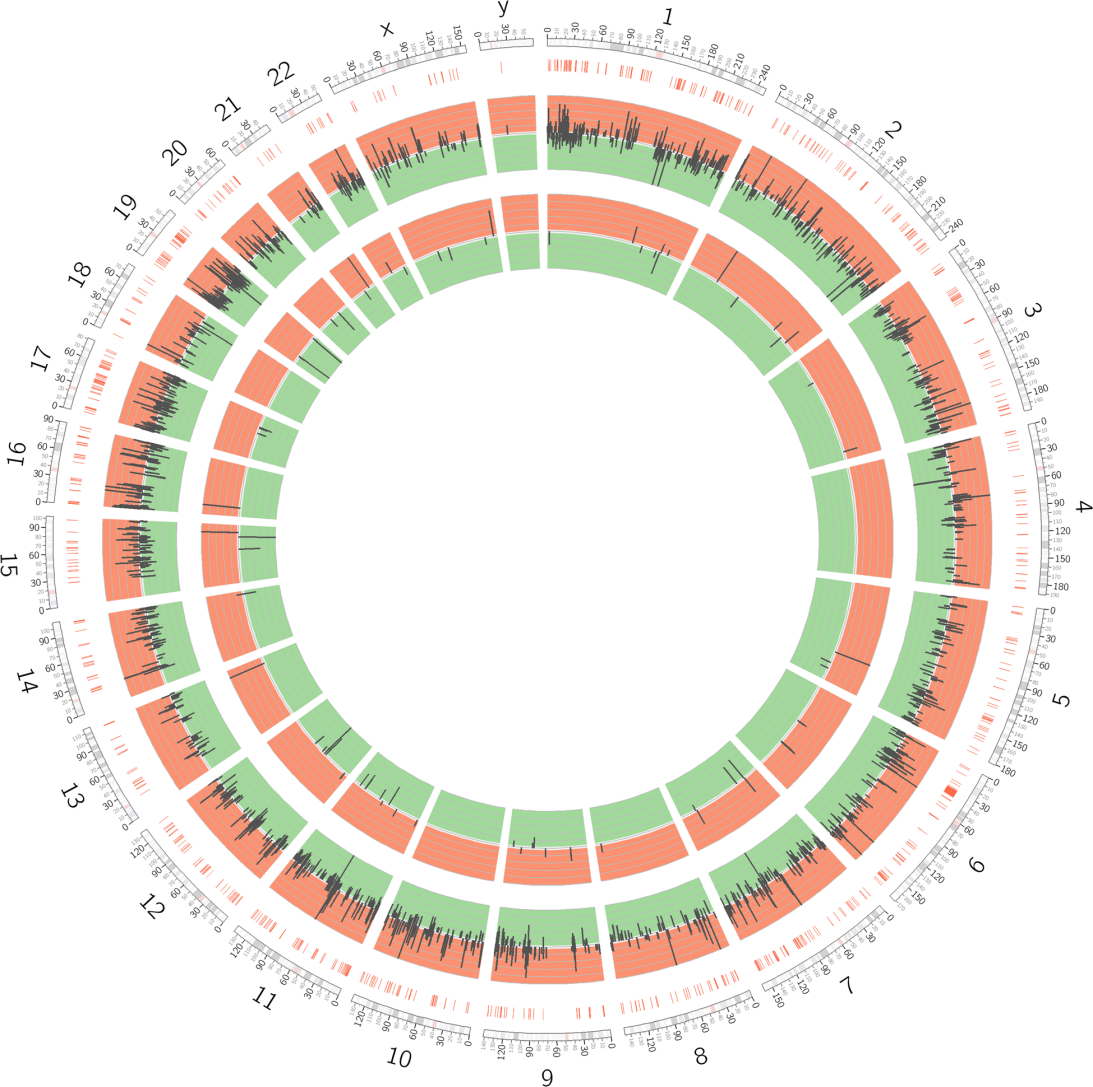
Circos plot incorporating differential gene and miRNA expression. Chromosome numbers and bands are identified in the outer-most ring. Other tracks from outer to inner represent: Chromosomal positions of genes implicated in PDAC tumorigenesis; differential mRNA expression (FDR < 0.05) between controls and PDAC (Up in PDAC: red, down in PDAC: green); differential miRNA expression (FDR < 0.05) between control and PDAC tissues.

From 921 sncRNAs with robust expression (Additional file 3: Table S3), 123 were significantly differentially expressed between the two conditions (Additional file 4: Table S4). Similar to MACE, sRNA-seq allowed a clear separation of cancer and control tissues by PCA, PCCs, and unsupervised clustering (Additional file 5: Figure S1). The twenty most significantly up-/downregulated sncRNAs are listed in Table 4. Among all differentially regulated sncRNAs, we found 104 mature miRNAs, 18 sdRNAs and one piRNA. An overview of differential RNA expression across the genome is given in Figure 2.

**Table 4:** **Significantly up- and downregulated sncRNAs**

**a) Downregulated in PDAC**					
**sRNA**	**NEV normal**	**NEV PDAC**	**Log2 FoldChange**	**FDR**	**Previously shown**
hsa-miR-216b	57,650.3	99.6	9.2	0.0000000	[7,8,11]
hsa-miR-216a-3p	3,218.9	4.1	9.6	0.0000000	[7,8,11]
hsa-miR-217	86,322.3	143.1	9.2	0.0000000	[7-9,11]
hsa-miR-216a-5p	35,959.9	144.7	8.0	0.0000000	[7-9,11]
hsa-miR-802	3,702.2	2.0	10.9	0.0000000	-
hsa-miR-148a-5p	4,252.9	178.9	4.6	0.0000000	[7-9,11]
hsa-miR-2114-5p	88.5	0.9	6.6	0.0000000	-
hsa-miR-375	121,790.3	11,992.2	3.3	0.0000001	[7-9,11]
hsa-miR-130b-5p	890.4	95.1	3.2	0.0000002	[7-9,11]
hsa-miR-148a-3p	224,094.2	25,764.7	3.1	0.0000005	[7-9,11]
hsa-miR-130b-3p	3,038.0	395.2	2.9	0.0000008	[7-9,11]
hsa-miR-190b	436.6	19.5	4.5	0.0000026	-
hsa-miR-2114-3p	44.7	0.3	7.0	0.0000270	-
hsa-sno-HBII-296B	1,159.5	208.8	2.5	0.0000525	-
hsa-miR-30c-2-3p	164.1	27.1	2.6	0.0000657	[7-12]
hsa-miR-219-5p	219.7	36.4	2.6	0.0000808	[7]
hsa-piR-017061	4,818,0	951,7	2,3	0.0001447	-
hsa-miR-30a-3p	1,350.4	275.0	2,3	0.0001447	[7-9,12]
hsa-miR-29c-3p	41,297.8	8.131.4	2,3	0.0003007	[7-9,12]
hsa-sno-U104	7,726.4	1.703.0	2,2	0.0007282	-
**b) Upregulated in PDAC**					
hsa-miR-135b-3p	1.2	80.9	−6.1	0.0000000	[11,12]
hsa-miR-135b-5p	948.9	9,475.0	−3.3	0.0000002	[11,12]
hsa-miR-21-3p	174.6	2,117.3	−3.6	0.0000005	[7-12]
hsa-miR-708-5p	309.2	3,100.3	−3.3	0.0000022	[7,10]
hsa-miR-615-3p	1.2	97.2	−6.3	0.0000024	[7]
hsa-miR-34c-5p	47.5	475.2	−3.3	0.0000048	[11]
hsa-miR-431-5p	7.0	99.7	−3.8	0.0000062	-
hsa-miR-511	17.1	242.6	−3.8	0.0000079	-
hsa-miR-143-5p	238.7	2,089.8	−3.1	0.0000092	[7-10,12]
hsa-miR-222-3p	2,263.7	17,399.2	−2.9	0.0000131	[7-12]
hsa-miR-34c-3p	2.7	49.7	−4.2	0.0000210	-
hsa-miR-155-5p	392.4	3,458.7	−3.1	0.0000210	[7-10,12]
hsa-miR-24-2-5p	396.5	2,965.3	−2.9	0.0000407	[9,10,12]
hsa-miR-34b-3p	1.0	29.5	−4.9	0.0000681	[7,9]
hsa-miR-708-3p	24.4	188.7	−3.0	0.0001073	[7,10]
hsa-miR-223-5p	17.4	204.8	−3.6	0.0001117	[7-10,12]
hsa-miR-34b-5p	12.5	96.7	−3.0	0.0001145	[7,9,11]
hsa-miR-10a-3p	101.1	643.8	−2.7	0.0001447	[7-10]
hsa-miR-196b-5p	10.1	738.0	−6.2	0.0001832	[9,11]
hsa-miR-2355-3p	3.8	43.2	−3.5	0.0002933	-

For the 20 most significantly up- and downregulated sncRNAs the mean normalized expression values (NEV) for each group is provided together with the ratio between normal and PDAC expression in log2 scale (log2FoldChange). In addition, the column “previously shown” indicates if other groups have provided evidence for the deregulation of the RNA before.

### Enrichment analysis of protein-coding genes for identification of biological and functional differences

Gene ontology (GO)-enrichment analysis of downregulated genes in PDAC revealed a total of 39 significantly enriched GO terms (Additional file 6: Table S5) across all three GO categories. Most of these terms were linked to normal pancreas function, as e.g. “Digestion” (FDR = 1E-5), “Steroid metabolic process” (0.002) or “Triacylglycerol lipase activity” (0.014). In addition, other GO terms corresponding to translation of mRNAs into proteins, as e.g. “Translational elongation” (5.6E-15), “Cytosolic ribosome” (3.2E-8) or “Ribosomal subunit” (2E-5) were enriched, indicating a loss of normal pancreas function.

Upregulated genes in PDAC were enriched in 208 GO terms (Additional file 7: Table S6), including “Immune response” (1.2E-17), “Cell proliferation” (4.45E-5) and “Cell migration” (9E-5). The second set represents an enhanced proliferative potential, the third a high metastatic potential of the cancer cells. The most significantly enriched GO term was “Extracellular matrix” (2.2E-30), corresponding to genes involved in fibrogenesis, such as collagens and fibronectin, as well as *TGFB* and genes related to this pathway. Several categories were related to a sustained angiogenesis, like “Vasculature development” (2E-8) or “Blood vessel development” (8E-8). Taken together, GO-analysis confirms a loss of normal pancreatic function in the tumor tissues, in combination with increased proliferative potential, extracellular matrix (ECM) activation, blood vessel formation, metastatic spread and the potential to escape the immune system.

### LncRNA expression in PDAC

Of 432 lncRNAs with robust expression, eleven were significantly up- and 32 downregulated in PDAC samples (Table 3). Other studies have already examined three lincRNAs (SNHG8, PVT1, H19), one NAT (HOTAIRM1) and two lncRNAs (MIAT, GAS5) which we identified as differentially expressed in PDAC [23]. Furthermore, the differential expression of two lincRNAs (LINC00261, LINC00152) [24] and two NATs (HNF1A-AS1, AFAP1-AS1) [25,26] were implicated in other cancer types.

### SncRNA expression analysis

Of 921 measured sncRNAs, 45 (30 miRNAs, fourteen snoRNAs, one piRNA) were significantly downregulated in PDAC tissues. Previous microarray or qPCR studies reported downregulation for 25 of these (Table 4). The most significantly deregulated sncRNA without previous implication in pancreatic cancer was miR-802, which was highly expressed in normal pancreas but not in PDAC tissues (log2fc = 11, FDR = 9E-29). Beside sdRNAs (e.g. a 34 bp fragment from sno-HBII-296B) and miRNAs, piR-017061, a piRNA located within the HBII-296A snoRNA, was significantly downregulated in PDAC compared to normal pancreas tissues (log2fc = 2.3, FDR = 0.0001).

A total of 78 sncRNAs (74 miRNAs, 4 sdRNAs) showed significant upregulation in PDAC. Several of these were previously implicated in pancreatic cancer development (e.g. miR-21, 143, 222, 155, 10a, 874) others have not been shown to be upregulated in PDAC before (e.g. miR-31, 511, 2355). The expression of all differential miRNAs is visualized in Additional file 8: Figure S2.

### *In silico* target analysis of miR-802

We used omiRas [27] to decipher potential interactions between miR-802 and genes significantly upregulated in PDAC, as detected by MACE. In total, 16 genes (*AMPD3, CDH11, IGFBP5, ITPR3, HOXA5, MMD, PGM2L1, SLC4A7, ST8SIA4, TCF4, TMEM92, TRIB2, TSHZ3, RAI14, ZFHX4, ZNF521*) were predicted to be upregulated due to loss of post-transcriptional silencing of miR-802 (for details see Additional file 9: Table S7). Enrichment analysis of miR-802 targets with starbase [28] revealed a significant enrichment of targets in Wnt signalling (p = 0.006), suggesting that loss of miR-802 might lead to increased Wnt activity in PDAC.

**Figure 3:**
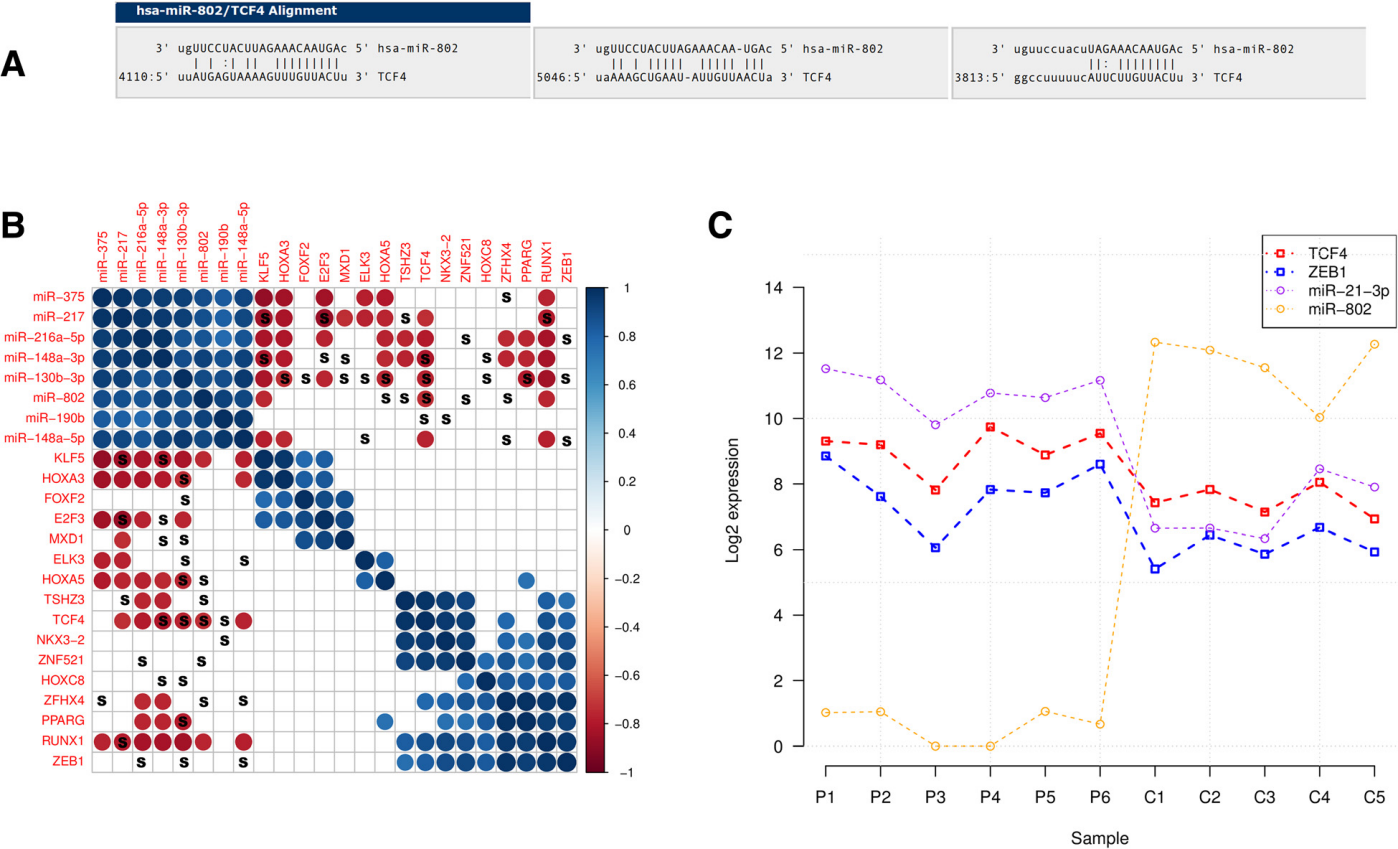
Co-expression of miR-802, *ZEB1*, *TCF4* and miR-21. **A)** Alignment of miR-802 to the predicted binding sites in the 3′ UTR of *TCF4*. **B)** Co-expression analysis of significantly upregulated transcription factors that harbour predicted miRNA binding sites in their 3′ UTRs for one of the ten most significantly upregulated miRNAs (miRNAs that have no seed sequence for a TF UTR not shown). Significant (p < 0.01) correlations are indicated by a dot, positive correlations are marked in blue, negative correlations in red. The more significant the correlation, the larger the dot size. Sequence complementarity between an UTR and a miRNA is indicated by an “S”. **C)** Expression of *TCF4*, *ZEB1* and miR-21 across all control (C) and PDAC (P) samples (significant positive correlation) as well as the expression of miR-802 (significantly inversely correlated). The normalized expression for each gene/miRNA is given in log2-scale for each sample.

The *TCF4* transcript (3.3-fold upregulated in PDAC, FDR = 0.001), encoding a transcription factor in the Wnt-signalling pathway, has the highest number of three mir-802 binding sites in its 3′ UTR (positions 3813, 4110, 5046, Figure 3A), and the interaction is predicted by all six interaction databases used for analysis. Co-expression analysis of all upregulated transcription factors (Additional file 10: Figure S3) revealed that the expression of *TCF4* is significantly correlated with *ZEB1* expression (PCC = 0.92, p = 5.2E-05). In addition, their expression is highly correlated with the expression of miR-21 (PCC = 0.88, p = 0.0003974) and inversely correlated with miR-802 expression (PCC = −0.83, p = 0.0015) (Figure 3B, C).

### Overexpression of miR-802 decreased TCF4 protein expression

**Figure 4:**
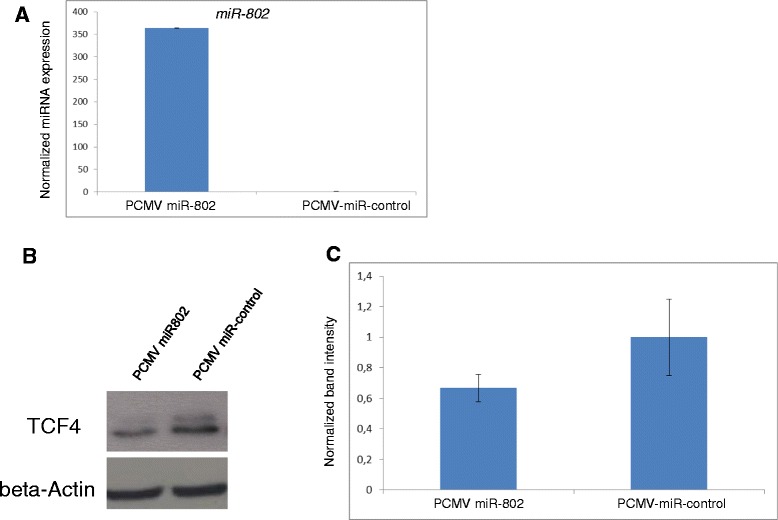
Re-expression of miRNA induces downregulation of TCF4. **A)** RT-qPCR of miRNA isolated from MiaPaCa cells 24 h after transfection with PCMV-MIR-802 or PCMV-MIR-Control. miRNA-802 expression: fold-change with standard deviation (n = 3). **B)** TCF4 expression was analyzed 24 h after expression of PCMV-MIR-802 and PCMV-MIR-Control in MiaPaCa cells. Β-Actin was used as a loading control and for normalization (n = 3). **C)** LI-COR quantification of TCF4 protein levels in miRNA-treated cells. TCF4 levels were normalized to β-Actin (n = 3).

Considering the absent expression of miR-802 in PDAC tissues and the *in silico* predicted binding sites of *TCF4*, we re-expressed miR-802 in MiaPaCa PDAC cells and assessed TCF4 expression. First, we induced miR-802 re-expression in MiaPaCa cells transfected with PCMV-MIR-802 (Figure 4a). Highly elevated levels of miR-802 were observed 24 h after transfection. To assess the effect of miR-802 on TCF4 protein levels, we harvested transfected MiaPaCa cells and analysed the proteins by western blot analysis (Figure 4b). Here, TCF4 protein levels decreased to 67% as compared with samples transfected with the negative control (Figure 4c).

### Validation of ZEB1 expression at the protein level

**Figure 5:**
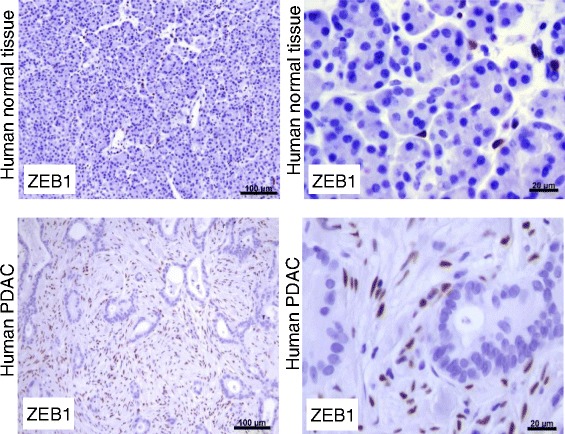
Immunohistochemistry of ZEB1 protein expression. Immunohistochemical detection of ZEB1 in human pancreatic tissue samples. Representative images of ZEB1 expression: Upper panels, ZEB1 is expressed in periacinar cells in normal pancreatic tissue samples. Lower panels, detection of ZEB1 in stromal cells, but not in epithelial tumor cells in PDAC samples.

Since we hypothesize that miR-802 regulates *ZEB1* expression *via TCF4*, we analysed expression levels of ZEB1 by immunohistochemistry in samples of human PDAC (n = 10) and normal pancreatic tissues (n = 10). In normal pancreatic tissue, ZEB1 was sparsely seen in periacinar cells (e.g. stellate cells). As depicted in Figure 5, we detected ZEB1 in PDAC samples in stromal cells within desmoplastic areas, but epithelial tumor cells did not express ZEB1. In accordance with previous observations [29] we detected ZEB1 in all analyzed pancreatic cancers only in the stromal compartment but not in epithelial cells. This observation emphasizes ZEB1 as a mesenchymal differentiation marker. We speculate that the newly identified miRNA802 might be involved in the regulation of ZEB1 and consequently might promote the mesenchymal character of pancreatic cancer.

### MiRNA-mRNA interaction network analysis of differentially expressed genes

**Figure 6:**
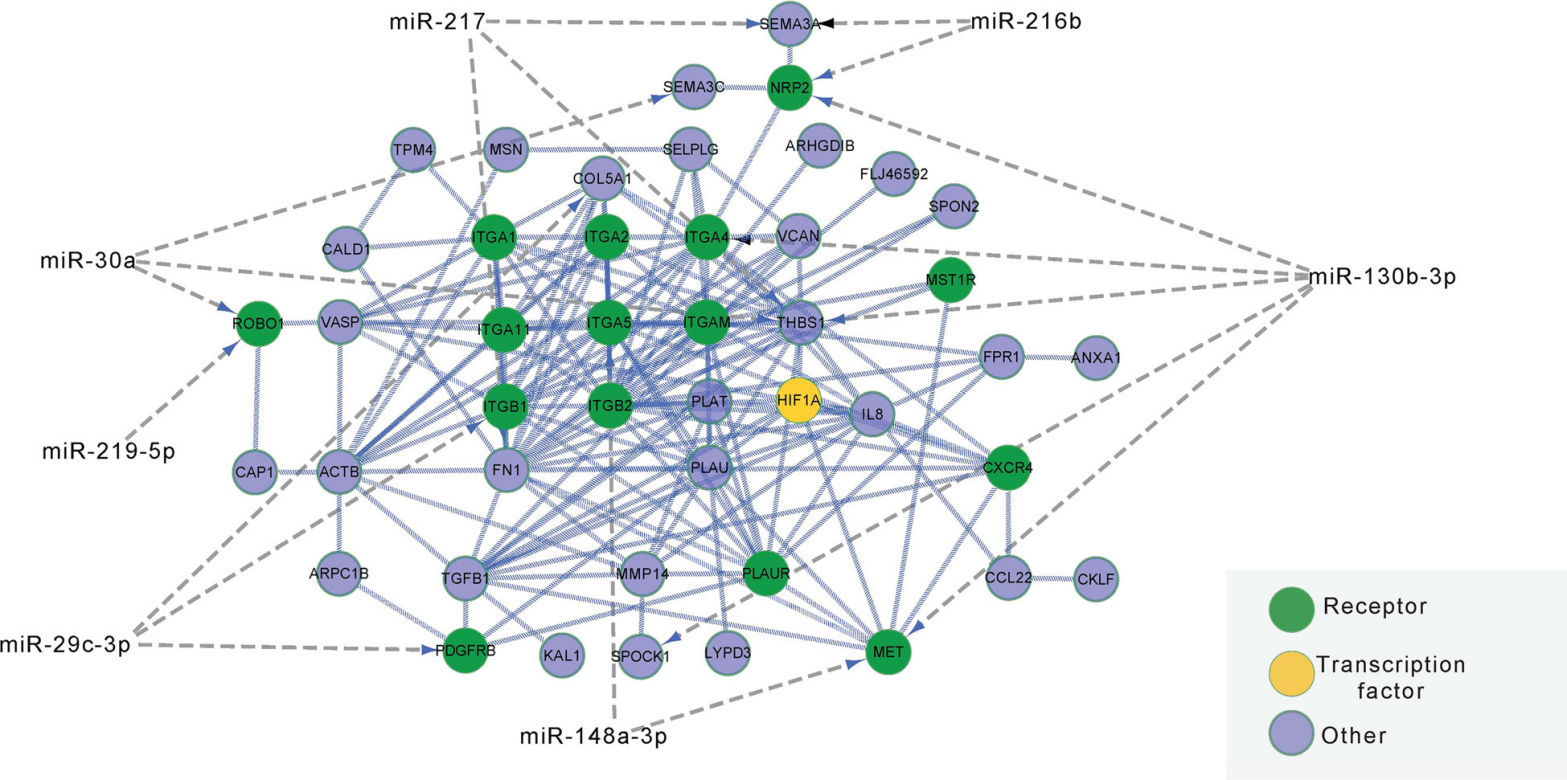
Influence of miRNAs on metastatic potential. Interaction network between gene products of upregulated genes from GO category: “Cell motion” and downregulated miRNAs in PDAC. Receptors are indicated in green, transcription factors in orange, and other proteins in blue. Predicted miRNA binding of the mRNAs encoding the protein is encoded by a grey line, protein-protein interactions by bold blue lines.

To test whether miRNAs are involved in tumor-specific functional categories detected by GO-enrichment analysis, we exemplarily created a miRNA-mRNA interaction network for the term “Cell motion” (Figure 6). The network contains interactions between gene products of upregulated transcripts in PDAC, as well as downregulated miRNAs whose loss might cause the upregulation of their target genes, predicted by at least three independent miRNA-mRNA interaction tools.

Of 63 differentially regulated genes involved in cell motion, 43 form a highly connected protein-protein interaction network with 164 connections.

From this network, twelve genes (*COL5A1, FN1, ITGA4, ITGB1, MET, NRP2, PDGFRB, ROBO1/SLIT, SEMA3A, SEMA3C, SPOCK1/SPARC, THBS1*) are connected to the loss of seven miRNAs (miR-29c-3p, 30a, 130b-3p, 148-3p, 216, 217, 219-5p). Five genes (*MET, NRP2, ROBO1/SLIT, SEMA3A, THBS1*) are under potential post-transcriptional control of two or more of these miRNAs. This crosstalk predicts miR-130b to regulate the expression of five genes from the network (*MET, SPOCK1/SPARC, THBS1, ITGA4, NRP2*).

### Confirmation of differentially expressed genes, lincRNAs and miRNAs by qPCR

**Figure 7:**
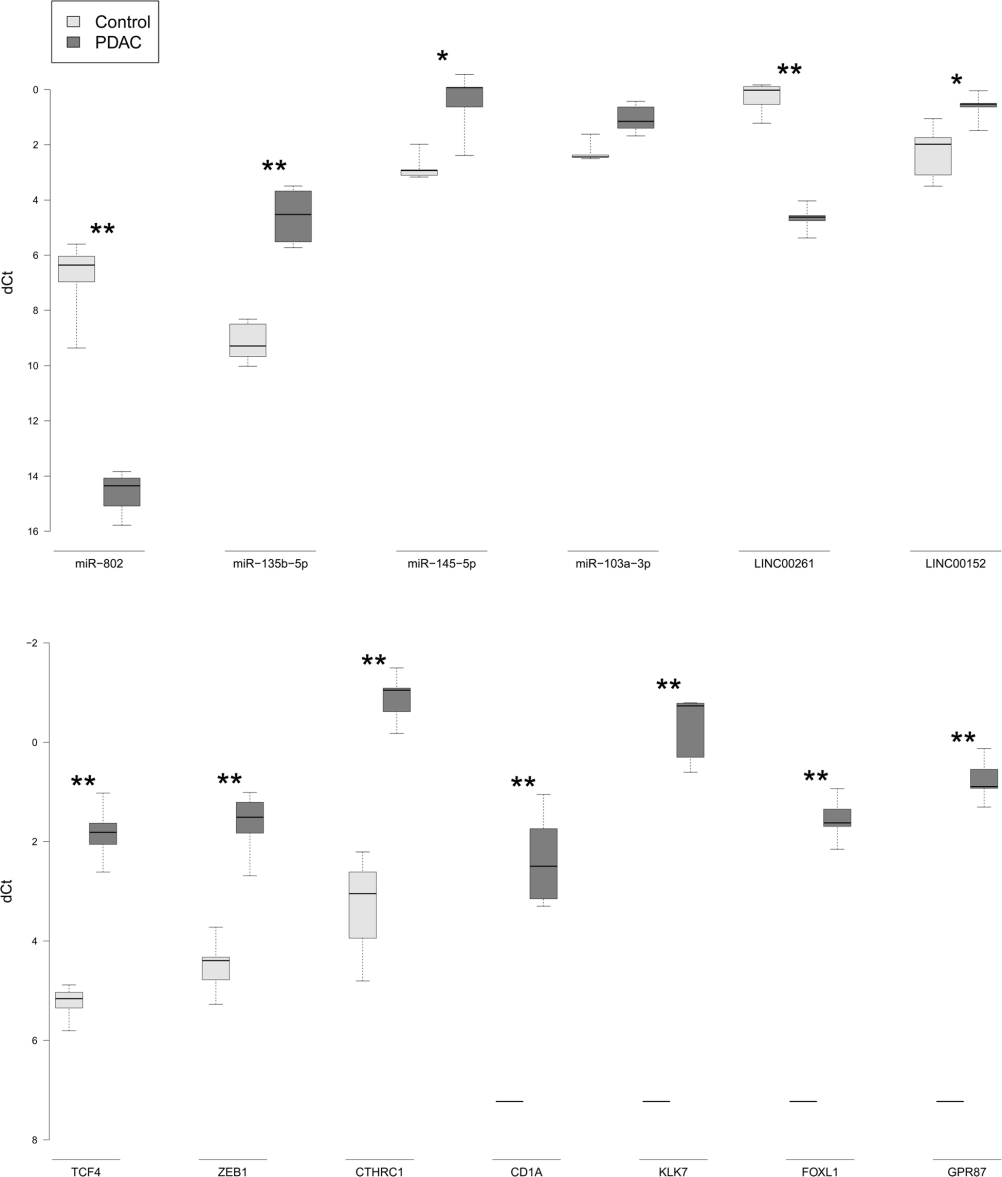
QPCR validation of sequencing results. The relative expression (Y-axis) for all candidate miRNAs/genes/lincRNAs (X-axis) is shown using boxplots for each condition. The bold black line represents the median expression across patients within a condition. *indictates a p-value < 0.05, **a p-value <0.01.

To validate sequencing results obtained by sRNA-seq and MACE, the expression of seven candidate genes, upregulated in PDAC (*TCF4*, *ZEB1*, *CD1A, FOXL1, GPR87, KLK7, CTHRC1*), one down- and three upregulated miRNAs (miR-802, 135b-5p, 145-5p, 103a-3p) as well as two lincRNAs (LINC00152, LINC00261) was verified by qPCR. The expression of *HPRT1* and miR-16 was used for normalization between both samples. The results of the qPCRs are given in Figure 7.

Except for miR-103a-3p, all tested RNAs were below a significance threshold of p < 0.05 (Wilcoxon’s rank sum test) when comparing expression of control and tumor samples. This is consistent with sequencing results, where similarly only miR-103a-3p did not reach the level of significance.

*CD1A*, *FOXL1*, *GPR87* and *KLK7,* mRNA expression was undetectable by qPCR in normal pancreatic tissue. This is consistent with sequencing results, where no or at most two reads (normalized) were annotated to these transcripts. Similar to sequencing, all four genes were robustly expressed in PDAC tissues, with C_t_ values ranging from 24.8 to 31.1 and a median C_t_ of 27.3.

Consistent with MACE results, *CTHRC1, TCF4* and *ZEB1* were significantly higher expressed in PDAC compared to normal pancreas tissues, with median normalized C_t_ (ΔC_t_) values between −1.8 and 1.8 in cancer- and 3.1-5.1 in normal tissues. The expression differences (log2 ratio normal/tumor tissue) measured by qPCR (negative ΔΔC_t_) highly agree with the log2 fold-changes of the sequencing results (PCC = 0.86).

Similarly, downregulation of miR-802 was validated by qPCR, with a median negative normal/tumor ΔΔC_t_ of 8.2 and p = 0 (Wilcoxon’s rank sum test), which is consistent with the log2fc and p-value estimated by sRNAseq (10.9, 0). Similar agreements between sequencing and qPCR results were obtained for the other three miRNAs.

Furthermore, the upregulation of LINC00152 (MACE log2fc: 2.3, qPCR: 1.5) and downregulation of LINC00261 (MACE log2fc: 5.3, qPCR: 4.4) in PDAC tissues was confirmed by qPCR.

### External validation of MACE data by microarray

Badea and colleagues [30] investigated 36 whole tumor- and adjacent normal pancreatic tissue samples by coding-gene microarray analysis. We compared the 53 most significantly upregulated genes in tumors from their publication with the MACE sequencing results based on logarithmic fold-change (Additional file 11: Figure S4) and statistical significance (p-value). Only five of the genes (*DCBLD1, PGM2L1, PDGFC, COX7A1, LY6E*), were not significantly upregulated (p < 0.05) in the MACE data, whereas 48 genes showed a significant upregulation as detected by both methods. The PCC between log2fc of MACE and microarray data (0.61) indicates a strong correlation between the results and underlines the reliability of the approach.

## Discussion

Our study investigated the coding- and non-coding transcriptomes of six PDAC patients and five healthy pancreatic control tissues. We detected 1,961 mRNAs, 43 lncRNAs and 123 sncRNAs as differentially expressed between the groups. Among these are several coding and non-coding RNAs without previous implication in pancreatic cancer development, most prominently miR-802 which is strongly downregulated in PDAC. Bioinformatic and functional analysis revealed post-transcriptional regulation of TCF4 protein levels by miR-802. Differential regulation of four miRNAS (miR-802, miR-135b-5p, miR-145-5p, 103a-3p), seven genes (*CD1A, FOXL1, GPR87, KLK7, CTHRC1, TCF4, ZEB1*) and two lincRNAs was confirmed by qPCR.

MiR-802 is the third most significantly repressed miRNA in PDAC, besides the tumor suppressor miRNAs miR-216 and miR-217 that - among others - target *KRAS*, *PTEN*, and *SMAD7* [29]. MiR-802 is mainly expressed in pancreatic acinar cells [31], which may be the cells of origin for pancreatic preneoplastic lesions and pancreatic cancer [32]. A significant downregulation of miR-802 is observed in mice with ethanol-induced chronic pancreatitis, which predisposes to pancreatic cancer [33]. In contrast, sRNA-seq of pancreatic cyst fluids from low-grade benign and high-grade invasive lesions revealed thirteen enriched miRNAs, among these miR-216, miR-217, and miR-802, in the cyst fluids derived from invasive carcinomas [29]. The reason for the inverse correlation between the expression levels of these tumor suppressor miRNAs in body fluids and tumors currently remains unexplained.

Since no previous studies have reported downregulation of miR-802 in pancreatic cancer, validated targets are rare. Nevertheless, miR-802 targets were identified in two other cancer-types: osteosarcoma and lung cancer [34,35]. In contrast to PDAC, miR-802 is upregulated in both cancers. MiR-802 elevation promotes proliferation of lung carcinoma cell lines by targeting the tumor suppressor gene *MEN1*. Similarly, cell proliferation was promoted by miR-802 in osteosarcoma, where the gene encoding p27, a negative cell-cycle regulator, is a direct target. In hepatocellular carcinoma miR-802 is more than 100-fold downregulated, but no targets have yet been identified [36].

Bioinformatic *in silico* prediction points to Wnt signalling related transcription factor TCF4 mRNA (~12-fold upregulated in PDAC, FDR = 5E-7) as a direct target of miR-802. To validate the generated *in silico* predictions, we re-expressed miR-802 in the PDAC cell line MiaPaCa and analysed TCF4 protein expression. After re-expression of miR-802, we observed a 30% reduction of TCF4, indicating a direct impact of miR-802 on TCF4 regulation.

TCF4 activates miR-21 transcription by direct binding to its promoter in epithelial cancer [37]. Other oncomiRs with TCF4 binding sites proximal to their promoter include miR-10a, miR-424, miR-935 and miR615 [38].

Furthermore, the regulation of miR-181a/b expression has been associated with TCF4 expression in hepatocellular carcinoma [38]. Consistent with these observations, our study validates the upregulation (4.6-79-fold, FDR 0.002-4.9E-7) of these six miRNAs with important functions in PDAC development [39,40] that have TCF4 binding sites in their promoter.

Additionally, Sanchez-Tillo and colleagues showed that TCF4 induces *ZEB1* (zinc finger E-box binding homeobox 1) transcription [41]. ZEB1 is an epithelial-to-mesenchymal transition (EMT)-activator that promotes PDAC tumorigenesis and metastasis [42]. *ZEB1* is, concordant to *TCF4*, 3.5-fold upregulated in PDAC in this study (FDR = 0.03). Consistent with these data, we detected ZEB1 protein expression in the mesenchymal compartment of all analysed PDAC samples.

To test whether specific miRNAs contribute to the metastatic potential of pancreatic cancers, we used omiRas to predict interactions between downregulated miRNAs and upregulated genes from the GO term “Cell motion”. The miRNA with the highest number of targets was miR-130b. Previous studies confirmed downregulation of miR-130b in pancreatic cancer and functional tests revealed that overexpression of miR-130b remarkably inhibited the invasiveness of pancreatic cancer cells [43]. MiR-130b loss (8-fold downregulated in PDAC, FDR = 8.2E-7) might lead to the upregulation of metastasis associated key oncogenes *MET, NRP2, ITGA4, THBS1* and *SPOCK1* [44,45]. Recently published data describe the impact of miR-130b in metastasis formation [46] and therefore validates the approach of *in silico* prediction of miRNAs.

In contrast to miRNAs, lncRNAs have just recently moved into the focus of cancer research [47,48]. Nevertheless, studies examining the role of lncRNAs in specific oncogenic processes are limited to date.

Liu and colleagues reported an increased expression of the lncRNA MALAT1 in PDAC compared to adjacent normal pancreatic tissues [49]. Furthermore, they described a significant correlation between MALAT1 expression levels and tumor size, tumor stage, invasion, and disease-free survival [49]. In our analysis, however, MALAT1 upregulation in PDAC was not found. This is further supported by the pancreatic expression database (PED), where MALAT1 was also not reported as differentially expressed [50].

Similar to MALAT1, an increased expression of HOX antisense intergenic RNA HOTAIR has been found in pancreatic tumors [51]. According to the PED, no significant upregulation of HOTAIR is found in PDAC tissues, which is consistent with our results. Overexpression of PVT1 in the pancreatic cancer cell line ASPC-1 resulted in decreased gemcitabine sensitivity [52]. In this regard, we found an approximately 6-fold upregulation of lncRNA PVT1 in pancreatic cancer.

Besides PVT1 we provide evidence for a deregulation of other 42 lncRNAs in PDAC. Among these LINC00152 is, like in PDAC, overexpressed in gastric cancer (GCC), and its high expression correlates with increased invasion [53]. Xia and colleagues speculate about LINC00152 functioning as a competing endogenous RNA (ceRNA) that sequesters miR-18a-5p, 195-5p, 139-5p and miR-31-5p in GCC and thereby influences *i.a. THBS1* expression [21]. We report LINC00152 overexpression in pancreatic cancer (5-fold, FDR = 7.1E-7), whereas all miRNAs with binding sites in the transcript are not significantly deregulated compared to control tissues. This indicates that increased LINC00152 expression might decrease the availability of particular miRNAs by competing for their binding and consequently lead to an upregulation of the miRNA target genes, such as *THBS1* (5-fold upregulated in PDAC, FDR = 7.7E-6).

Overexpression of snoRNAs is a common feature in breast and prostate cancer [15], and contributes to tumorigenicity *in vitro* and *in vivo*. SnoRNA U50 is downregulated in prostate cancer and potentially functions as a tumor suppressor in other cancer types [54]. Several reports describe that snoRNAs are further processed to generate smaller fragments (sdRNAs) with miRNA-like functionality [55]. Currently, there is no evidence for snoRNAs/sdRNAs involved in pancreatic cancer development. To our best knowledge, this is the first report of differential regulation of snoRNAs/sdRNAs in PDAC. The most significantly regulated sdRNA (34 bps long) is from sno-HBII-296B (SNORD91B), which is approximately 5-fold downregulated in PDAC (FDR = 5.2E-5). However, its functional role and that of other differentially expressed snoRNAs/sdRNAs remains unclear; similarly, the functions of piRNAs has not been fully elucidated. PiRNAs are usually involved in germline development, silencing of transposons, and maintenance of DNA integrity [56]. Upregulated expression of piR-651 has been described in several cancer cell lines, where it promotes cell growth and might serve as a marker for cancer diagnosis [56]. Here we report the downregulation of piR-017061 in PDAC, a piRNA that is located within the sno-HBII-296A snoRNA.

## Conclusion

This study underlines the role of miRNAs in PDAC and provides evidence for differentially regulated miRNAs that have not been previously implicated in PDAC. Additionally, we provide evidence that novel sncRNA classes, snoRNAs and piRNAs are differentially regulated between normal pancreas and PDAC tissues. Using a bioinformatics approach, we connect mRNA sequencing data with miRNA expression to assign potential functions to miR-802 and other miRNAs. Furthermore, we provide evidence for the differential expression of a variety of lncRNAs in pancreatic cancer.

## Methods

Tissue samples were obtained from six patients with PDAC who underwent resection at the Department of Surgery, Technical University of Munich, Germany.

Normal pancreatic tissue samples from five patients without pancreatic ductal adenocarcinoma were used as controls.

Tissue collection was approved by the Ethics Committee of the Technical University of Munich and informed consent was obtained from all patients. Tissue were collected directly in the operating theatre and were immediately stored at −80°C until analysis.

### Isolation of RNA

20 mg of frozen tissue were disrupted and homogenized (TissueLyser, Qiagen) and RNA was isolated (NucleoSpin miRNA Kit, Macherey-Nagel) in two fractions (small RNA < 200 nt and large RNA > 200 nt).

### Preparation of small RNA libraries

For preparation of small RNA libraries, 5 μg RNA (small RNA fraction) was size-selected (<40 nt) by polyacrylamide gel electrophoresis (FlashPAGE, Life Technologies) and precipitated. About 30 ng small RNA (<40 nt) was successive ligated (T4 RNA Ligase 1 and T4 RNA Ligase 2, NEB) to modified 3′ and 5′ adapters (TrueQuant RNA adapters, GenXPro). Adapter-ligated RNA was reverse transcribed (SuperScript III, Life Technologies) and amplified by PCR (KAPA HiFi Hot-Start Polymerase, KAPA Biosystems). Amplified libraries were size-selected by polyacrylamide gel electrophoresis (PAGE) and sequenced (HiSeq2000, Illumina).

### Preparation of massive analysis of cDNA ends (MACE) libraries

MACE libraries were prepared as described by Müller et al. [57]. Briefly, poly-adenylated RNA was extracted (Dynabeads mRNA Purification Kit, Life Technologies) from 5 μg RNA (large RNA fraction) and reverse transcribed (SuperScript Double-Stranded cDNA Synthesis Kit, Life Technologies) with biotinylated poly(dT) primers. cDNA was fragmented (Bioruptor, Diagenode) to an average size of 250 bp. Biotinylated ends were captured by streptavidin beads (Dynabeads M-270 Streptavidin Beads, Life Technologies) and ligated (T4 DNA Ligase 1, NEB) to modified adapters (TrueQuant DNA adapter, GenXPro). The libraries were amplified by PCR (KAPA HiFi Hot-Start Polymerase, KAPA Biosystems), purified by SPRI beads (Agencourt AMPure XP, Beckman Coulter) and sequenced (HiSeq2000, Illumina).

### Cell culture and transfection

The pancreatic cancer cell line MiaPaCa was maintained at 37°C in a humified atmosphere of 5% CO2 and 95% air in Dulbecco’s modified Eagle Medium (Life Technologies, Inc, Darmstadt, Germany). The cells were transfected with Lipofectamine2000 (Life Technologies, Inc, Darmstadt, Germany) according to the manufacturer’s protocol with either PCMV-MIR-802 or PCMV-MIR-Control (OriGene, Rockville, USA).

### RT-qPCR

MiRNA was extracted from MiaPaCa cells with the miRNeasy Mini Kit (Qiagen, Hilden, Germany). Reverse transcription was performed using the RevertAidH Minus First Strand cDNA Synthesis Kit (Thermo Scientific, Braunschweig, Germany) using specific hsa-miR-802 and as control hsa-miR-16 primer. Amplification of cDNA was performed using the TaqMan Small RNA Assays Applied Biosystems (Life Technologies, Inc, Darmstadt, Germany). The primers for the cDNA synthesis and for the TaqMan analysis were included in the kit from TaqMan Small RNA Assays Applied Biosystems (Life Technologies, Inc, Darmstadt, Germany). For quantification of miRNA-802 expression the results were normalized against miR-16 expression.

### Western blot

For quantification of protein levels after miRNA expression, transfected MiaPaCa cells were fractionated using the NE-PER Nuclear and Cytoplasmic Extraction Reagents (Thermo Scientific, Braunschweig, Germany). 20 μg of protein from the nuclear fraction was loaded onto a 10% polyacrylamide gel and was then electrophoretically transferred to a nitrocellulose membrane. The membrane was blocked with Tween-20 (0.05%)-TBS (pH 7.4; 0.1 M Tris Base, 1.4 M NaCl) containing 5% milk, followed by incubation with respective primary antibody α-TCF4 (LS-Bio, Eching, Germany) at a concentration of 1:1000 or as a control α-β-Actin (Abcam, Cambridge, UK) with a concentration of 1:2000. Membranes were washed with Tween-20 (0.05%)-TBS and were incubated with a horseradish peroxidase (HRP)-conjugated anti-rabbit antibody (1:5000). Signals were detected using the enhanced chemiluminescence system (ECL, Amersham Life Science Ltd., Bucks, UK). Films were scanned with a CanoScan 9900F scanner (Canon, Japan). Protein levels were quantified using the Odyssey software LI-COR and normalized to the β-Actin control.

### Confirmation of MACE results by qRT-PCR of selected genes

LincRNA and mRNA expression analysis was carried out with the QuantiTect Multiplex PCR Kit (Qiagen) in combination with Superscript III reverse transcriptase (Life Technologies) and PrimeTime qPCR Assays (IDT). For miRNA detection, we used the miRCURY LNA Universal RT microRNA PCR system (Exiqon) according to the recommendations of the manufacturer. Reverse transcription and PCR-amplification for mRNA expression studies were performed with 50 ng of the large total RNA fraction. All quantitative real-time PCR reactions were carried out on the Lightcycler 480 II (Roche). For mRNAs/lincRNAs the expression of housekeeping gene *HPRT1* was measured for data normalization, while miR-16 served as endogenous control for miRNA quantification. Differential expression between control and tumor tissues was assessed using the ∆∆C_t_ method, p-values were calculated with Wilcoxon’s rank sum test.

### Immunohistochemistry analysis

Immunohistochemistry was performed using the Dako Envision System (Dako Cytomation GmbH, Hamburg, Germany). Consecutive paraffin-embedded tissue sections (3 μm thick) were deparaffinized and rehydrated using routine methods. Antigen retrieval was performed in citrate buffer (pH 6.0; 10 mM citric acid) in a microwave oven for 10 minutes. Endogenous peroxidase activity was quenched by incubation in TBS (pH 7.6; 0.2 M Tris Base; 1.4 M NaCl) containing 3% hydrogen peroxide at room temperature for 10 minutes. After permeabilization with 0.5% TritonX, nonspecific reactivity was blocked with TBS containing 5% BSA. Sections were incubated with the ZEB1 antibody (ZEB1: Atlas Antibodies #AMAb90510 (1:400)) at 4°C overnight followed by incubation with horseradish peroxidase-linked goat anti-mouse antibody, followed by a color-reaction with diaminebenzidine and counterstaining with Mayer’s hematoxylin.

### Bioinformatics analysis of MACE data

To remove any PCR-bias, all duplicate reads detected by the TrueQuant technology were removed from the raw datasets. The remaining reads were additionally quality trimmed and the poly(A)-tail was clipped off. After raw data processing, reads were aligned to the human genome with novoalign (http://www.novocraft.com). Annotations for genomic mapping positions were derived by the RefSeq annotation track that includes coding genes as well as lncRNAs (http://genome.ucsc.edu/cgi-bin/hgTables) and only uniquely mapped reads were taken into account. Normalization and test for differential gene expression between normal and tumor tissue were calculated using the DESeq R/Bioconductor package [58]. To account for multiple testing, the false discovery rate (FDR) was estimated. Genes with FDR *< 0.05* and *|log2fc| > 1.6* were considered as differentially expressed.

### Bioinformatics analysis of small RNA-seq data

The sRNA-seq data was quantified and tested for differential expression with omiRas [27]. Briefly, for each small RNA-seq library, data processing started with 3′ adapter clipping by a local alignment of the adapter sequence to each read. Subsequently, Illumina’s marked quality region was removed and the reads were summarized to UniTags. Singletons were removed from the data set and the remaining tags were mapped to the human genome (hg19) with bowtie. The mapped tags were annotated with the help of various models of coding and non-coding RNAs retrieved from the UCSC table browser. Tags mapping to exonic regions of coding genes were excluded from further analysis. Non-coding RNAs were quantified in each library. For tags mapping to multiple genomic loci the number of reads corresponding to the tag was divided by the number of mapping loci. Normalization and test for differential expression was performed in the same way as described for mRNAs.

### Evaluation of normal pancreas function in control libraries

To underline the usability of apparently normal pancreas tissue samples to serve as healthy controls, all genes were sorted in ascending order according to their normalized mean expression in normal sequencing libraries. To determine genes encoding pancreas specific proteins, functional annotations of the fifty most highly expressed genes were extracted from genecards [59].

### Enrichment analysis

Differentially expressed genes were uploaded to the Database for Annotation, Visualization and Integrated Discovery (DAVID) resource [60] using an enrichment cutoff of FDR < *0.05*.

### Co-expression analysis

A list of all human transcription factors (TFs) was received from AnimalTFDB [61]. Individual expression values of significantly upregulated (in PDAC) transcription factors were clustered using the k-means method with PCC as a distance measure and six initial clusters. The median expression of all transcription factors for each sample was taken as the representative expression for the cluster. To detect the influence of significant miRNA loss on transcription factor upregulation, PCCs were calculated for each TF/TF, miRNA/miRNA and miRNA/TF pair.

### Network analysis

Interactions between miRNAs and transcripts with negatively correlated differential expression were detected with omiRas, using a minimum database overlap of three required interaction databases. Additionally, the STRING database v 9.0.5 [62] was used with standard parameters to detect interactions between gene products of differentially expressed genes.

## Availability of supporting data

The data sets supporting the results of this article are available in the ArrayExpress expository, accession E-MTAB-3494.

The data sets supporting the results of this article are available in the GEO repository, [GEO: will be inserted after publication].

## Additional files

Additional file 1: Table S1. Expression of all mRNAs and lncRNAs detected with MACE. Additional file 2: Table S2. Differentially expressed mRNAs and lncRNAs between controls and PDAC tissues. Additional file 3: Table S3. Expression of all sncRNAs detected with sRNA-seq. Additional file 4: Table S4. Differentially expressed sncRNAs between control and PDAC tissues. Additional file 5: Figure S1. NGS sncRNA profiles discriminate PDAC from healthy control tissues. Unsupervised hierarchical cluster analysis with Euclidean distance measure of differentially expressed sncRNAs clearly separates healthy controls and diseased patients. (B) Principle component analysis (PCA) of all sncRNAs from the eleven examined samples. (C) Pearson product–moment correlation coefficient (PCC) for all samples compared within the control and patient group as well as between both groups. Additional file 6: Table S5. Enriched GO terms of significantly downregulated genes in PDAC. Additional file 7: Table S6. Enriched GO terms of significantly upregulated genes in PDAC. Additional file 8: Figure S2. Boxplot of differential miRNAs for control and PDAC libraries. For each differentially expressed miRNA (FDR < 0.05) the normalized expression in log2 scale for each group is visualized as a boxplot. Additional file 9: Table S7 Predicted, significantly upregulated targets of downregulated miR-802. Additional file 10: Figure S3 Transcription factor co-expression analysis. The normalized expression of differentially upregulated transcription factors is given for each library in each of the eight clusters determined by k-means clustering with PCC as a distance measure. The median expression in each cluster is indicated by a bold black line, the expression for the transcription factors in the cluster is indicated by different colours. Additional file 11: Figure S4 Comparison of MACE and Microarray data. For 53 genes (colored dots) the log2 fold-change between control and PDAC tissues as determined by microarray (X-axis) and NGS-based MACE (Y-axis) is given. The blue line represents the linear regression line.

## Abbreviations

PDAC: Pancreatic ductal adenocarcinoma
MACE: Massive Analyis of cDNA Ends
sncRNA: Small non-coding RNA
miRNA: microRNA
sdRNA: small nucleolar-derived RNA
piRNA: Piwi-interacting RNA
lncRNA: Long non-coding RNA
lincRNA: Long intergenic non-coding RNA
NAT: Natural antisense transcript
ceRNA: Competitive endogenous RNA
sRNA-seq: Small RNA-Sequencing
qPCR: Quantitative real time PCR
PCA: Principal component analysis
PCC: Pearson’s moment product correlation coefficient
TCF4: Transcription factor 4
GO: Gene ontology
ZEB1: Zinc finger E-box binding homeobox 1
GEO: Gene Expression Omnibus
FDR: False discovery rate
DAVID: Database for Annotation, Visualization and Integrated Discovery
TF: Transcription factor
