# Erratum to: Next-generation sequencing reveals novel differentially regulated mRNAs, lncRNAs, miRNAs, sdRNAs and a piRNA in pancreatic cancer

**DOI:** 10.1186/s12943-015-0401-6

**Published:** 2015-07-31

**Authors:** Sören Müller, Susanne Raulefs, Philipp Bruns, Fabian Afonso-Grunz, Anne Plötner, Rolf Thermann, Carsten Jäger, Anna Melissa Schlitter, Bo Kong, Ivonne Regel, W Kurt Roth, Björn Rotter, Klaus Hoffmeier, Günter Kahl, Ina Koch, Fabian J Theis, Jörg Kleeff, Peter Winter, Christoph W Michalski

**Affiliations:** Molecular BioSciences, Goethe University, Frankfurt am Main, Germany; GenXPro GmbH, Frankfurt Biotechnology Innovation Center, Frankfurt am Main, Germany; Department of Surgery, Klinikum Rechts der Isar, Technische Universität München, Munich, Germany; Molecular Bioinformatics Group, Institute of Computer Science, Cluster of Excellence Frankfurt ‘Macromolecular Complexes’ Faculty of Computer Science and Mathematics, Frankfurt am Main, Germany; Department of Surgery, University of Heidelberg, Heidelberg, Germany; GFE Blut mbH, Frankfurt Biotechnology Innovation Center, Frankfurt am Main, Germany; Institute of Computational Biology, Helmholtz Zentrum Munich, Neuherberg, Germany; Department of Mathematics, TU Munich, Boltzmannstrasse 3, Garching, Germany; Department of Pathology, Klinikum Rechts der Isar, Technische Universität München, Munich, Germany

## Erratum

Due to a publisher error, this article [1] was originally published with an incorrect table 3. The correct table can now be found at http://tinyurl.com/mvkzay8.
