# Supplementary figures and images for: Next-generation sequencing reveals novel differentially regulated mRNAs, lncRNAs, miRNAs, sdRNAs and a piRNA in pancreatic cancer
Source: Mol Cancer. 2015 Apr 25;14:94. doi: 10.1186/s12943-015-0358-5 (PMC4417536; doi:10.1186/s12943-015-0358-5)

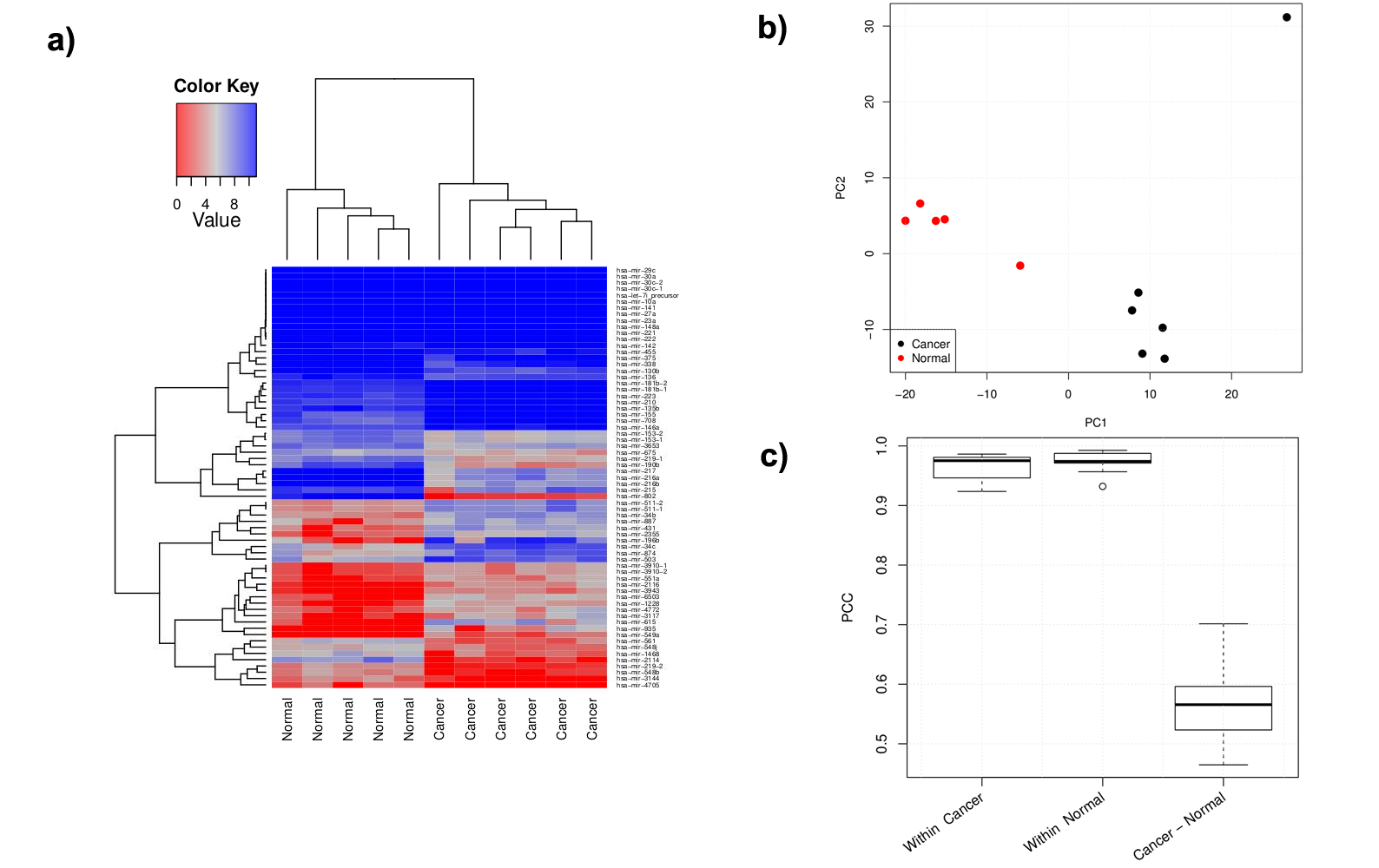

Supplement: Additional file 5: Figure S1. — NGS sncRNA profiles discriminate PDAC from healthy control tissues. Unsupervised hierarchical cluster analysis with Euclidean distance measure of differentially expressed sncRNAs clearly separates healthy controls and diseased patients. (B) Principle component analysis (PCA) of all sncRNAs from the eleven examined samples. (C) Pearson product–moment correlation coefficient (PCC) for all samples compared within the control and patient group as well as between both groups. [file 12943_2015_358_MOESM5_ESM.tiff]

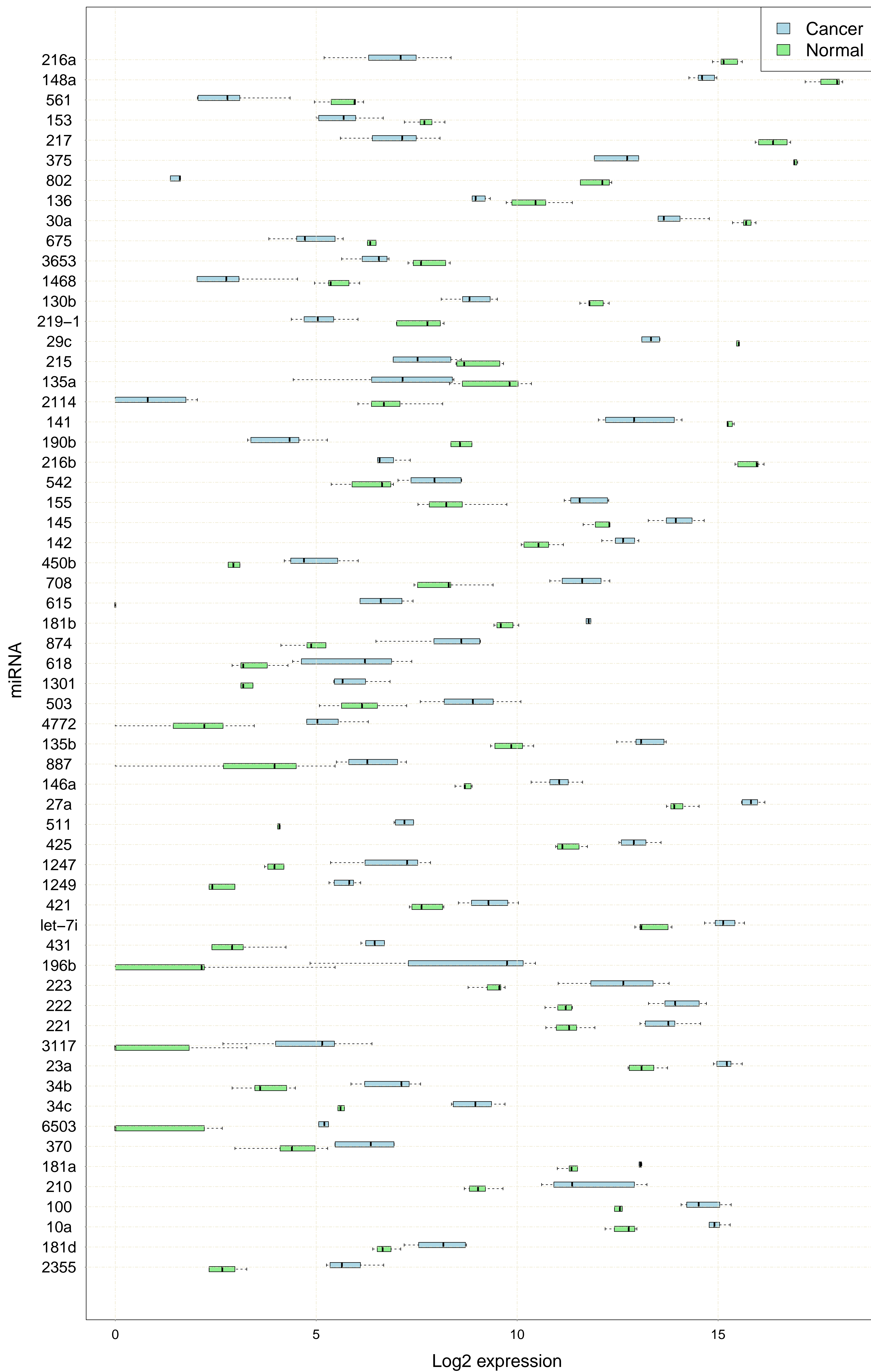

Supplement: Additional file 8: Figure S2. — Boxplot of differential miRNAs for control and PDAC libraries. For each differentially expressed miRNA (FDR < 0.05) the normalized expression in log2 scale for each group is visualized as a boxplot. [file 12943_2015_358_MOESM8_ESM.pdf]

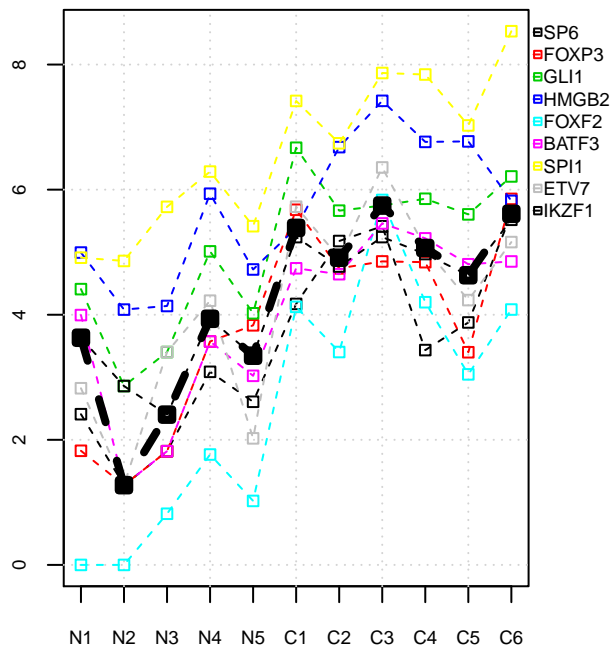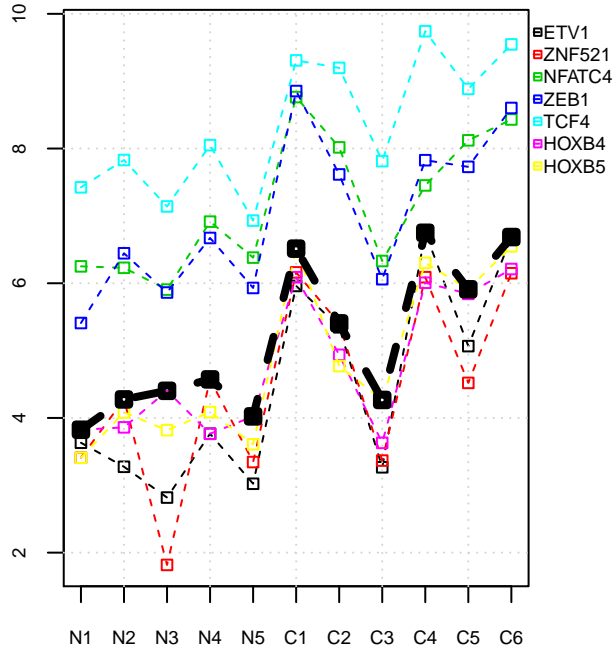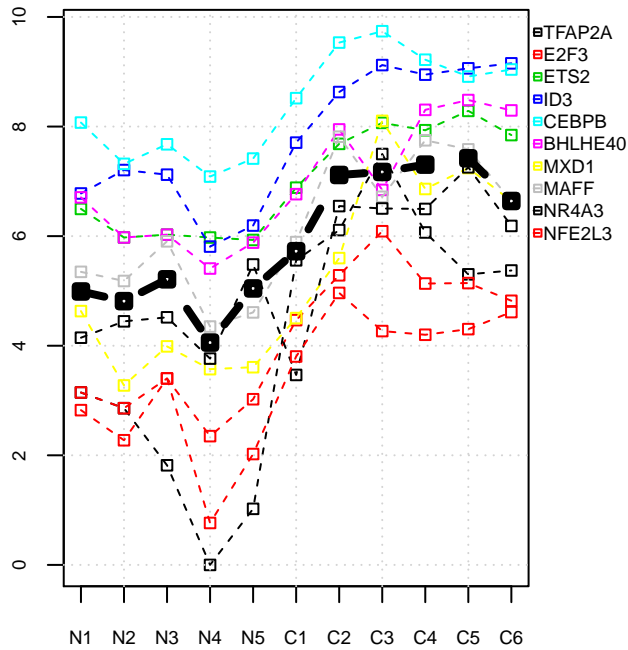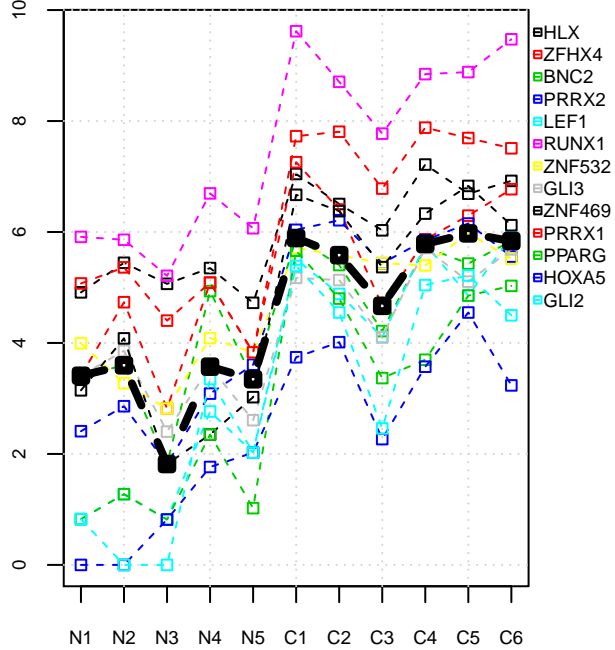

Supplement: Additional file 10: Figure S3 — Transcription factor co-expression analysis. The normalized expression of differentially upregulated transcription factors is given for each library in each of the eight clusters determined by k-means clustering with PCC as a distance measure. The median expression in each cluster is indicated by a bold black line, the expression for the transcription factors in the cluster is indicated by different colours. [file 12943_2015_358_MOESM10_ESM.pdf]

# Comparison: Microarray vs. MACE

cor = 0.61

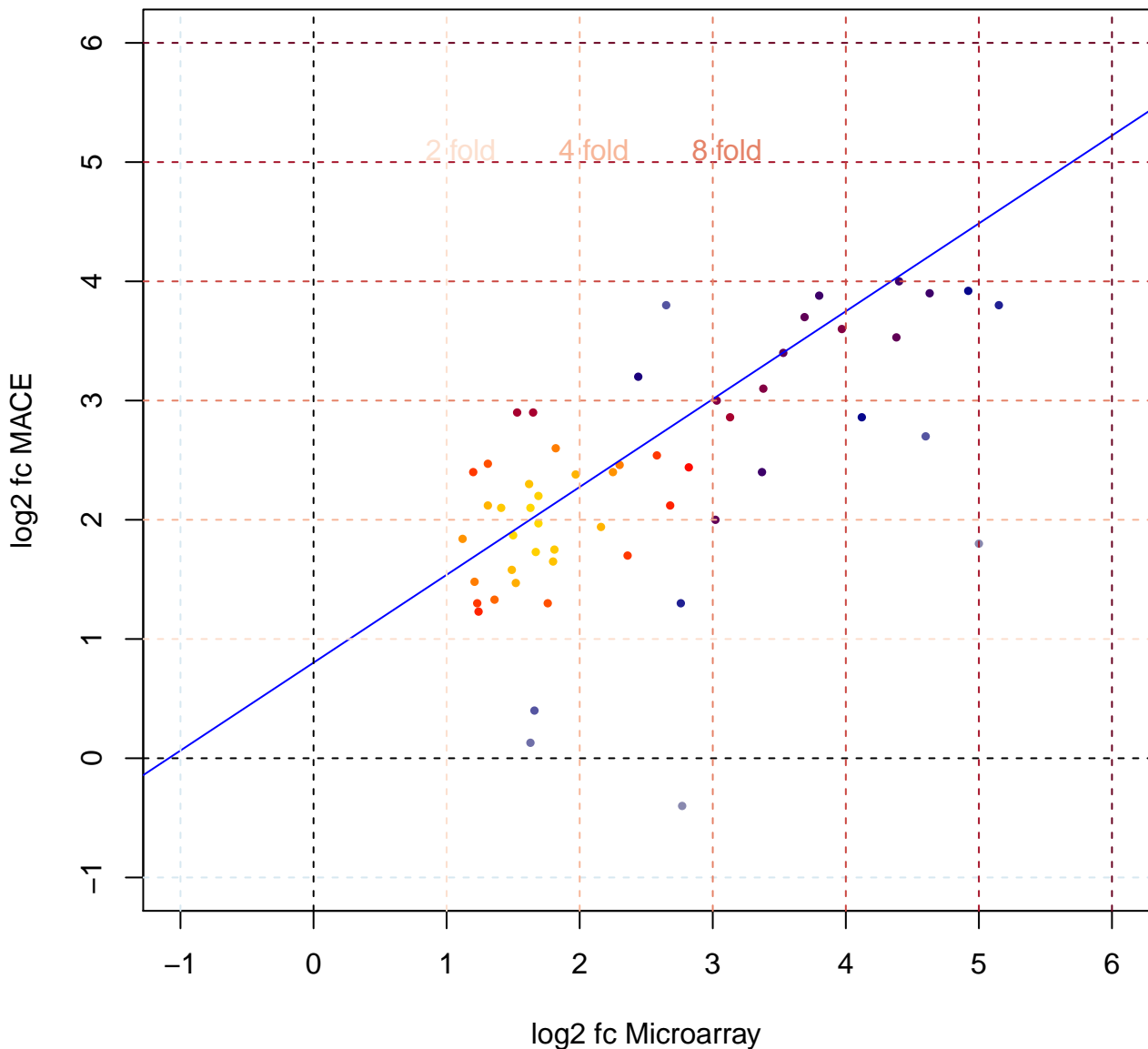

Supplement: Additional file 11: Figure S4 — Comparison of MACE and Microarray data. For 53 genes (colored dots) the log2 fold-change between control and PDAC tissues as determined by microarray (X-axis) and NGS-based MACE (Y-axis) is given. The blue line represents the linear regression line. [file 12943_2015_358_MOESM11_ESM.pdf]
